# Polyphenols in Foods and Their Use in the Food Industry: Enhancing the Quality and Nutritional Value of Functional Foods

**DOI:** 10.3390/ijms26125803

**Published:** 2025-06-17

**Authors:** Nurten Coşkun, Sümeyye Sarıtaş, Mikhael Bechelany, Sercan Karav

**Affiliations:** 1 Department of Molecular Biology and Genetics, Çanakkale Onsekiz Mart University, Çanakkale 17000, Türkiye; nnurten.coskun@gmail.com (N.C.); sumeyyesaritas@stu.comu.edu.tr (S.S.); 2 Institut Européen des Membranes (IEM), UMR 5635, University Montpellier, ENSCM, CNRS, F-34095 Montpellier, France; 3 Functional Materials Group, Gulf University for Science and Technology (GUST), Masjid Al Aqsa Street, Mubarak Al-Abdullah 32093, Kuwait

**Keywords:** polyphenols, functional food, antioxidant activity, valorization

## Abstract

Polyphenols are known as secondary metabolites, which are crucial bioactive compounds that play a significant role in enhancing human health. Chromatographic methods are typically used to identify polyphenols after food extraction. The extraction methods are fundamental, however, they are implemented with some differences, including extractant type, according to the food. Polyphenols are mostly found in some foods, including grapes, olives, cherries, and apples. Foods have diverse polyphenols, which differ according to the food type. Moreover, they have flavonols, flavanols, flavones, flavanones, isoflavones, and anthocyanins as various subgroups of polyphenols, which can change in terms of quantity and quality along with several factors, including the type, growing region, germination time, and harvest season of the food. The consumption of polyphenols is crucial for human health due to their anti-cancer, anti-tumor, anti-inflammatory, cardiometabolic risk management, antimicrobial, immunomodulatory, and antioxidant effects. In the valorization of polyphenols, the consumption dose is also important to effectively benefit from the polyphenols of plant-based foods. Several in vitro and in vivo studies have tested the polyphenols’ digestion ability and preservation ability in gut microbiota and their effect on the microbiota to determine the benefits and effects of polyphenols in several areas. According to these studies, polyphenols can be used to fight against disease. In addition, diverse applications, including encapsulation and polyphenol coating, are used to stabilize, preserve, and improve the bioaccessibility of polyphenols. Even though polyphenol-rich foods are consumed for nutrition in daily life, they are also used as nutritional ingredients in the food industry to produce functional foods, and functional foods are enriched with food by-products to enhance their nutritional value, especially in terms of polyphenols. Particularly, food by-products are used to enrich functional foods, which are preferred in healthy life diets due to the diversity and amount of bioactive ingredients, including the polyphenol types of the food by-products. Furthermore, polyphenols also provide the preservation ability of storage and improve the bioaccessibility of bioactive ingredients during the digestion of functional foods. This review article examines the polyphenol ingredients of several types of food used in the food industry. It explains the effective factors that affect the amount and type of food and determines the impact of polyphenols on polyphenol-enriched products and functional foods. The article also provides a brief exemplification of the value of polyphenol-rich food by-products in the context of functional food production. Several studies presented in this review article demonstrate the value of polyphenols, particularly in the food industry and functional food production.

## 1. Introduction

Polyphenols are crucial plant-based phenolic compounds that include aromatic rings and hydroxyl groups in their conformation, and they have several disease-preventing properties, including antioxidant activity [1]. They are used in several fields, including the food industry, pharmacology, and medicine [2,3]. Flavonoids, phenolic acids, polyphenolic amides, lignans, stilbenes, tannins, and curcuminoids are varieties of polyphenols [4,5]. These varieties have subtypes, such that the flavonoids have six subtypes involving flavonols, flavanols, flavones, flavanones, isoflavones, and anthocyanins, which are most frequently detected in most foods [6,7]. Specifically, some foods, including onions, grapes, cherries, and apples, have a high polyphenol content [8,9]. They are used in the food industry due to their health-beneficial effects [10,11].

### 1.1. Common Health-Promoting Properties of Polyphenols

Polyphenols have diverse properties, including cardiometabolic effects, antimicrobial effects, anti-inflammatory effects, antioxidant activity, anti-cancer effects, anti-tumor effects, anti-viral effects, and prebiotic and immunomodulatory activity, essential for human and other living health [12,13]. There are several polyphenol consumption-related health studies [14,15,16]. In a study, catechin, a polyphenol obtained from green tea, exhibited a preventive property in obesity and related diseases, including hypercholesterolemia and hyperglycemia, when in vivo studies were applied to rats [17]. Moreover, polyphenols improve cardiometabolic risk management to avoid the risks of obesity and cholesterol by limiting insulin and cholesterol [17]. In another study examining polyphenols from fruits, extracted polyphenols from raspberries were used together with prebiotic fructooligosaccharides to prevent nonalcoholic fatty liver disease by avoiding the accumulation of fats in the liver of Zucker rats [18]. Furthermore, apple polyphenols improved gut microbiota health and decreased appetite by preventing the unstable weight gain of the mice in vivo studies by applying a diet that used a high simple carbohydrate diet with the polyphenols of the apple [19]. In another in vivo study, apple polyphenols saved the rats from neural injury, which was induced by chronic ethanol exposure [20]. Its polyphenols, especially phloretin, demonstrate antimicrobial and anti-inflammatory effects on the respiratory pathogens that cause chronic obstructive pulmonary disorders, which are bacterial-induced problems [21].

Blueberries were used to obtain different polyphenols and determine diverse properties, including anti-inflammatory, antioxidant activity, and miRNA regulation or inhibition ability of the polyphenols, with several methods, including 2,2-Diphenyl-1-picrylhydrazyl (DPPH) assays for antioxidant activity [22]. Even though several polyphenols were identified in the study, phenolic acid was the most frequently detected polyphenol group. Phenolic acids provide the health effects of blueberries, including antioxidant activity. However, in miRNA regulation, other compounds of the blueberries without phenolic acids were effective.

Plant-based polyphenols can be combined with polyphenols obtained from other plants to enhance the health effects of the polyphenols [23]. In a study, blackberry polyphenols were combined with tea polyphenols to improve the oxidative stability of lard and olive oil [24]. The content of blackberry anthocyanins and the effect of different antioxidants on the acid degrees of lard and olive oil were determined. In addition, a comparison of the anti-lipid-oxidant efficiency of several antioxidants and antioxidant capacity was detected by detecting the scavenging capacity of DPPH-free radicals and 2,2’-Azino-bis(3-ethylbenzothiazoline-6-sulfonic acid) cation radicals. This study shows the antioxidant capacity of blackberry polyphenols.

### 1.2. Enhancement of Polyphenol Nutritional Value Mixed with Another Food

Polyphenol-rich foods can be combined with other ingredients to create healthy and enriched products [25,26,27]. In the food industry, polyphenol-rich foods are dried to use as ingredients in the production of functional food, but the amount of bioactive compounds in polyphenol-rich foods can degrade during drying [28]. As an example, blueberries were dried to enhance the nutritional value of black tea [28]. In the study, iron-polyphenol complex formation, polyphenol profile, antioxidant activity, and sensory properties of the blueberry-added black tea were negatively affected by drying. Despite the degradation of bioactive compounds of blueberries by drying, black tea was nutritionally enhanced with blueberries. Also, these results not only came from drying but were also affected by fruit concentration and infusion temperature.

### 1.3. Common Extraction Methods for Polyphenols

Diverse extraction methods and processes are employed to obtain polyphenols, depending on the composition and ingredients of the food [29,30]. Blueberries were extracted using natural deep eutectic and conventional solvents as extractants in a study [31]. The most successful solvents were determined to be natural deep eutectic solvents due to the high extraction capacity of the total phenolic content, total flavonoid content, total anthocyanin content, anti-radical activity, reducing power, and metal chelating activity of the blueberries. In another study, different extraction solvents were used, and optimum conditions were determined to obtain high levels of antioxidant phenolic compounds and antioxidant activity in strawberry fruits [32]. The solvents that have different polarities were used to determine the best solvent for the fruit extraction, and the best usable solvent was chosen as acetone for strawberries to obtain polyphenols. Also, optimum extraction conditions containing extraction time, temperature, and liquid/solid ratio were detected. Furthermore, the optimum extraction conditions were determined for raspberry leaves with different extraction methods or extractants, and the study also determined that steam explosion pretreatment was suitable to improve the accessibility of the polyphenols of the food [33]. As a result, diverse extraction methods are used to efficiently obtain polyphenols from foods.

### 1.4. The Importance of the Consumption Dose of Polyphenols

The consumption dose of polyphenols is an important determinant in demonstrating the diverse properties of polyphenols, including antioxidant, antimicrobial, and anti-inflammatory effects [34,35]. In a study applied to the rats, three different doses of blueberries were fed to rats, and dose-dependent polyphenol concentrations were obtained. In the rats’ colons after different dose intakes, a high amount of cinnamic and hippuric acids and a low amount of phenolic acids, flavonols, and anthocyanins were obtained according to the dose of blueberry polyphenols. Also, some polyphenols among these arrived at saturation degrees while different doses of polyphenols were taken. In another study, saturated fat was applied to rats with polyphenol intake [36]. In conclusion, cecal microbiota activity, lipid metabolism, and inflammation degrees were diverse when two different doses of raspberry polyphenols were taken. While lipid metabolism was provided with two different doses, the health-promoting effect of the polyphenols was obtained with the high doses fed.

### 1.5. Several Methods for Preserving Polyphenols During Digestion and Storage

Polyphenols may be degraded or inhibited in terms of their bioavailability during digestion or storage [37]. Different methods are used to preserve polyphenols, including encapsulation and microencapsulation. Encapsulation is a method to improve the stability and bioaccessibility of polyphenols in food [38]. In a study, strawberry juice was encapsulated by freeze-drying, which is a drying method that uses pea protein and okra mucilage as an encapsulating material [39]. At the end of the study, the polyphenols of strawberries and their antioxidant activity were preserved, and the bioaccessibility and stability of the polyphenols were increased. In another study, green tea was encapsulated by spray drying to enhance the efficiency assessment of the polyphenols. Another encapsulation method, microencapsulation, is an application to increase the shelf life of foods, especially in the food industry [40]. It can be used to preserve the bioactive ingredients of foods, including polyphenols. In a study, green tea leaf extract was obtained by supercritical fluid extraction and then encapsulated by spray drying, which used carrier materials such as maltodextrin, gum arabic, and chitosan with different ratios [40]. The encapsulation was applied due to the sensitivity of green tea to environmental conditions with high temperature, pH, and oxygen. As a result, the encapsulation efficiency was 71.41–88.04% for the total phenolic content and 29.52–38.05% for the antioxidant activity. It also preserved the total catechin, total phenolic content, and antioxidant activity at a temperature below 25 °C.

In addition to the encapsulation methods, polyphenol-rich food products are used to preserve other foods by using these products as packaging material [41]. In a study, chitosan-based apple peel polyphenols were used as a protective material for strawberries by coating, and they preserved the strawberries during the storage period [42]. The coating was prepared by applying chitosan, which was dissolved in acetic acid solution (1.0%, *v*/*v*) and stirred, then glycerol (30%, *w*/*w*) was added to the solution. Additionally, apple peel polyphenols were integrated into the solution. After that, the strawberries were disinfected with a 2% NaClO solution and dried slowly. After these, prepared strawberries coated with the solution containing the apple polyphenols were drained and dried. Finally, the strawberries were packaged in polypropylene plastic trays and stored at 20 °C. In conclusion, the application preserved the strawberries in terms of bioactive compounds, and it decreased the degradation of the properties and ingredients of the food.

**Figure 1:**
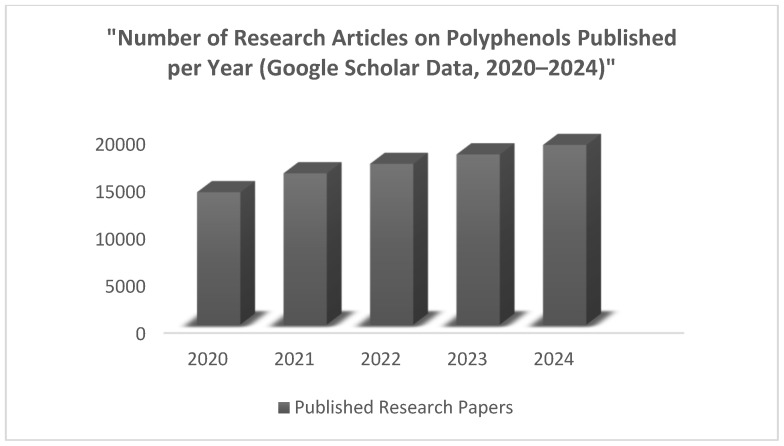
Number of Research Articles on Polyphenols Published per Year (Google Scholar Data, 2020–2024).

Furthermore, several studies are present in the literature on the polyphenolic ingredients of food (Figure 1). The keyword of the research for polyphenols in the Google Scholar platform is ‘Plant-based polyphenols’. The graph illustrates the evolution of the number of research articles during the 2020–2024 period. The data from 2025 are not included in this graph. There are several different studies that deal with polyphenols, but the polyphenol topic is very crucial for future studies, especially in pharmacology, medicine, or the functional food industry. Also, more studies are required for this topic.

**Figure 2:**
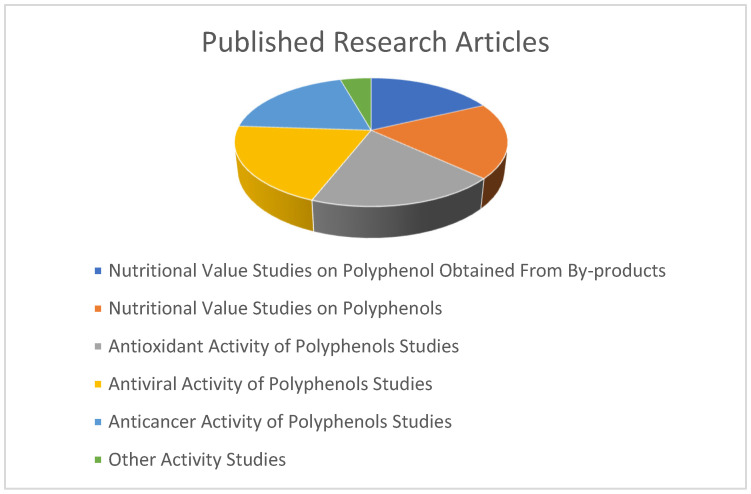
Distribution of published research articles on various polyphenol topics on Google Scholar over the past years.

Polyphenols have several nutritional values and health-promoting effects, including antioxidant activity, antiviral activity, and anticancer activity (Figure 2) [43]. Additionally, the polyphenols can be extracted from by-products. This is very important for the economy. The graph demonstrates that recent research articles have focused on certain topics about polyphenols. In the graph, the research articles about nutritional value studies of foods and their by-products, their antioxidant activity, antiviral activity, and anticancer activity are shown.

## 2. Polyphenols in Foods

There has been an enhanced demand for research and consumption of functional foods [44,45,46]. Food polyphenols are used in several industries, including medicine and the food industry, thanks to their diverse effects and properties, like anti-cancer and anti-inflammatory actions [47,48]. These are antimicrobial properties, antioxidant activity, anti-inflammatory effects, anti-cancer effects, etc. [49,50]. When compared with other food ingredients, food polyphenols exhibit a variety of polyphenol types, including anthocyanins and flavonoids [51,52]. Also, diverse plant materials, several extraction methods, and solvents are used to obtain diverse polyphenol compounds (Table 1) [53]. The extraction solvent is determined by the type of polyphenols and their polarity [54]. Flavonols and flavones are medium-polar compounds, and suitable solvents for the extraction can be ethanol, methanol, and ethanol–water because of the term ‘polar dissolve polar’ [55]. Flavan-3-ols (catechins) and tannins are polar, and they are extracted with water, ethanol, and methanol as extractants. Anthocyanins and phenolic acids are highly polar, and water, ethanol, and acidic solvents are used as extractants in their extraction [56]. Their polarity properties determine the affinity of polyphenols to extractants and the success of the extraction.

Several different methods and solvents similar to each other are applied to obtain polyphenols from fruits [82]. In a study, blueberries were freeze-dried, and their polyphenols were extracted and determined [67,83]. Firstly, blueberries were frozen with liquid nitrogen, and freeze-dried powder was obtained. Then, acidified methanol (0.3% HCl [*v*/*v*]) was used as the main extractant, which was used to wash the powder, and the extractant was evaporated. Anthocyanin was obtained with the lower layer separation of the previous process, ethyl acetate addition, supernatant separation, supernatant drying, powder production, and freeze drying. Also, an AB-8-type macroporous adsorption resin and the eluent were especially used to purify the anthocyanins. Blueberry polyphenols were obtained with partially different pathways. Initially, dry blueberries were washed with anhydrous ethanol and filtered to completely mix polyphenols and extractants. After that, the extracted liquid was evaporated, and the dried substance was washed with petroleum ether to remove the lipids. Then, ethyl acetate was added, and other future pathways were the same as anthocyanin extraction and purification, except for the eluent type of purification. The identification of polyphenols was performed with high-performance liquid chromatography and mass spectrometry. At the end of the study, delphinidin-3-glucoside, quercetin 3-O-galactoside, pelargonidin-3-O-galactoside, malvidin-3-O-glucose, phenylpropanoid compound chlorogenic acid isomers, flavonoid substance epicatechin gallate, and kaempferol-3-rhamnoside were especially obtained. Additionally, the antioxidant activity, antitumor activity, and immune function of anthocyanins and other polyphenols were studied and characterized. From another perspective, the solvent or extractant used can demonstrate differences even in the same plant material. For instance, in research on blueberries, acetone was used as an extractant, different from the previous study [81]. Also, in this study, the total polyphenol fraction, anthocyanin-enriched fraction, and proanthocyanidin-enriched fraction of blueberries were applied separately using different solvents. At the endpoint, these polyphenols’ antimicrobial and anti-inflammatory effects were studied. In a study about blackberry polyphenols, several polyphenols, including anthocyanins, cyanidin-3-O-glucoside, cyanidin-3-O-rutinoside, ellagitannins, catechins, etc., were extracted with an extraction method using 70% acetone and acidified methanol (the methanol: HCl ratio was 99:1) as extractants [84]. Flash chromatography and mass spectrometry were used to identify polyphenols, and the antioxidant activity of polyphenols was determined and searched in this study.

Furthermore, blueberries have antioxidant, antimicrobial, anti-inflammatory, and anticancer activities due to their polyphenol contents [85]. In another study, blueberries’ antitumor effects and immunomodulatory activities were detected, and these properties came from the polyphenols of blueberries [86]. Polyphenols can increase the health of microbiota [87]. In mice, blueberry polyphenols exhibited prebiotic activity and prevented obesity by improving fat metabolism and remodeling the gut microbiota, especially in the fecal-stage mice [88]. Fecal stage mice refer to the mice that are used to collect fecal samples in the fecal stage to study gut microbiota. Also, blueberry polyphenols, including those extracted from fruit and leaves, inhibit the neuroinflammatory response in microglia, and they can be preventive for neurological diseases, including Parkinson’s and Alzheimer’s disorders [89]. Furthermore, the number of polyphenols can exhibit an alternative according to the part of the food [90]. Polyphenol concentrations obtained from different parts, including seed, pulp, and the whole fruit of a red raspberry, demonstrated diversity. In this study, different polyphenol concentrations affected the different organisms’ improvement in microbiota, and also, the high-fat diet-induced obesity was balanced and inhibited by polyphenols.

Moreover, in another study, strawberry polyphenols, including anthocyanin and proanthocyanidin, were extracted with 70% ethanol and detected, and the antioxidant activity of these polyphenols was determined [91]. The detection was acquired using the pH differential method for the total monomeric anthocyanin content, high-performance liquid chromatography-diode array detection for anthocyanins, and the Folin–Ciocalteu method for the total phenolic content. Polyphenols in black raspberries were detected in another study [59]. Methanol, acetone, and ethanol with 30% water were used as extractants to obtain polyphenols, and ultra-high-performance liquid chromatography quadrupole time-of-flight mass spectrometry/mass spectrometry was used to identify polyphenols. As a consequence, various polyphenols containing cyanidin-based anthocyanins, multiple ellagitannins, free ellagic acid, gallic acid, 3,4-dihydroxybenzoic acid, 4-hydroxyphenyl acetic acid, ferulic acid, rutin, and quercetin were detected, and their health-promoting activity on gut microbiota was determined in an in vitro study. Polyphenols were also detected in apple fruits [19]. The effect of apple polyphenols on the microbiota and appetite was studied. The ameliorated high-carbohydrate diet-induced body weight gain of the mice was decreased with polyphenol consumption by regulating the microbiota and appetite. Additionally, date fruits are also polyphenol-rich fruits that show particularly antioxidant activity, and they have been used as potential nutraceuticals and in functional food production to enhance the nutritional value [91]. They have diverse polyphenolic compounds including gallic acid, coumaric acid, manisic acid, tannins, myricetin, and epicatechin [92].

In a study about artichokes, which is a vegetable, methanol was used as an extractant, and high-performance liquid chromatography/electrospray ionization tandem mass spectrometry was applied to identify their polyphenolic contents [75]. Various polyphenols, including luteolin, luteolin-O-glycoside, luteolin-7-O-rutinoside, apigenin, etc., were obtained and characterized. Additionally, their hepatoprotective activity was searched and determined. In a study, polyphenols of Welsh onion (*Allium fistulosum*) leaves were extracted with 70% *v*/*v* ethanol [64]. The total phenolic and anthocyanin contents, especially cyanidin and quercetin-3-glucoside, were obtained by applying ultra-high-performance liquid chromatography–electrospray ionization positive mode-orbitrap mass spectrometry analysis, and their antioxidant activity was determined. Red onions were extracted to obtain their polyphenols, including benzoic acid, rosmarinic acid, quercetin, rutin, pyrogallol, ρ-coumaric acid, quercetin, and quercetin [60]. They were extracted by ultrasound- and enzyme-assisted extractions using 80% ethanol and identified by high-performance liquid chromatography analysis. In addition, the antioxidant activity of their polyphenols was even detected. In another study, *Hettiarachchi* et al. determined the total phenolic content and total flavonoid content, including gallic acid, quercetin-3-glucoside, quercetin-3-rhamnoside, and myricetin-3-galactoside, of eggplant and spinach varieties, and their antioxidant activity was detected [76]. This result was obtained with methanolic extraction by methanol/water (80%, *v*/*v*) as an extractant, and their total phenolic and flavonoid contents were identified by the Folin–Ciocalteu method and a spectrophotometric method.

Legumes, including black beans and chickpeas, contain several polyphenolic compounds [93,94]. In a study, black beans were used to obtain polyphenols, which were studied to determine their antioxidant activity and anti-aging potential [68]. The extractant of this study was ethanol-water (50:50 *v*/*v*) in supercritical fluid extraction. Folin–Ciocalteu’s method was used for the total polyphenols, the pH differential method (AOAC Official Method 2005.02) for total anthocyanins, and electrospray ionization mass spectrometry analysis to identify phenolic compounds, including malvidin-3-glucoside, cyanidin-3-glucoside, delphinidin-3-glucoside, petunidin-3-O-β-glucoside, catechin, delphinidin 3-glucoside, myricetin, and sinapic acid. Chickpea hull, as a legume, was extracted with acetone, water, and acetic acid (70:29.5:0.5, *v*/*v*/*v*) [69]. Ultra-high-performance liquid chromatography was used to identify the polyphenols, and gallic acid and rutin were especially determined. Also, the antioxidant and anti-inflammatory potentials of the polyphenols were determined.

Nuts and seeds are rich in polyphenols, and they can be used in several functional foods due to their beneficial properties for humans [95,96]. In a study, the walnuts were searched to obtain polyphenols and extracted with 100% hexane (1:10 *w*/*v*) [57]. The Folin–Ciocalteu method was used to determine the total polyphenol content, and the polyphenol contents of the nuts were identified by reverse-phase high-performance liquid chromatography and high-resolution Fourier transform mass spectrometry. In conclusion, ellagic acid, strictinin, 3-methoxy-5,7,3′,4′-tetrahydroxy-flavone, gallic acid, ellagic acid pentoside, etc., were determined, and these polyphenols showed the inhibitory effect on the human intestinal glucose transport, human α-glucosidase activities, and human salivary and pancreatic α-amylases. In another study, the polyphenols of hazelnut skin were identified, and their antioxidant activity was detected [63]. Hazelnut skins were roasted before extraction by pure ethanol was applied, and several types of polyphenols, including gallic acid, protocatechuic acid, catechin, epicatechin, and quercetin, were identified with high-performance liquid chromatography. Also, the total polyphenolic content was determined by spectrophotometry, and the antioxidant activity of specified polyphenols was detected. According to a study, pecan polyphenols inhibit the enzyme activity that deals with starch digestion [65]. In the survey, acetone/deionized water/acetic acid (70:29.5:0.5, *v*/*v*/*v*) at a ratio of 6:10 (*w*/*v*) solution was used as the extractant, and the total phenolic content was detected by Folin–Ciocalteu’s method. Furthermore, in vitro studies were applied for scratch digestion, and the polyphenols of pecan controlled blood glucose. In addition, flax (*Linum usitatissimum* L.) seeds were used to obtain polyphenols, and the antidiabetic and anti-inflammatory effects of the polyphenols were determined in a study [97]. For the extraction of the seeds, 70% methanol was applied, and liquid chromatography with tandem mass spectrometry analysis identified the polyphenols of the seeds, including oleocanthal, oleuropein, hesperetin, ursolic acid, amentoflavone, quercetin-3-*O*-glucoside, quercetin-3-*O*-glucuronic acid, kaempferol-3-*O*-glucose, quercetin-3-*O*-hexose-deoxyhexoside, etc. For this research, in vitro and in vivo experiments were applied, and the effects of polyphenols were determined. Particularly, some enzyme activities, including α-amylase inhibitory and α-glucosidase inhibitory activities, were detected.

Additionally, herbs and spices have polyphenols, and these are used in several functional foods due to their various properties [98]. In a study, the antioxidant and antibacterial activity of clove (*Syzygium aromaticum*) and thyme (*Thymus vulgaris*) extracts were determined [77]. Overall, 95% ethyl alcohol was used as an extractant. The Folin–Ciocalteu method was applied to determine the total phenolic compounds, and the aluminum chloride colorimetric method was used to determine the total flavonoid compounds of the clove and thyme. Another food in this part is star anise. Star anise (*Illicium verum*) was extracted with distilled water, and high-performance liquid chromatography was used to identify polyphenols including gallic acid, 4-Hydroxybenzoic acid, catechin, chlorogenic acid, caffeic acid, syringic acid, vanillic acid, *p*-Coumaric acid, salicylic acid, rutin, etc. [66]. Also, the antioxidant, anti-obesity, and hypolipidemic effects of the polyphenols were detected in the research with a high-fat–sugar diet-induced obesity rat model. In another study, oregano leaves were extracted with ethanol (100%), and the bioactive components like polyphenols were identified by gas chromatography–mass spectrometry; the total polyphenol content was detected with the Folin–Ciocalteu method; and the total flavonoid content was determined with the process based on aluminum chloride [73]. Also, antioxidant activity and the internal and immunobiological effects of oregano bioactive ingredients, especially polyphenols, were determined. In another study, the polyphenols of sweet basil leaves (*Ocimum basilicum* L.) were extracted with 70% ethanol, and phytochemical analysis was applied to detect secondary metabolites, including tannins and flavonoids as polyphenols [74]. The Folin–Ciocalteu method for the total polyphenol content and the process based on aluminum chloride for total flavonoid content were utilized.

## 3. Several Influencing Factors on the Polyphenol Content of Foods

Several parameters, including the growing region, seasons, maturity, and obtained stage of plant-based food, determine polyphenol quantities and types [97,99]. The polyphenol concentration can be changed with the foods’ ripening and maturity stages, and the polyphenolic contents can increase during maturation [100]. The growth process of the food was divided into several parts. The highest level of the polyphenols, especially flavan-3-ol derivatives, was determined in the early improvement stage of the food. In conclusion, the polyphenol quantity of the food decreased toward the end of the ripening. In a study, the maturity period of the blackberries was divided into three stages [101]. The total phenolic content and total flavonoid content diminished, while the total anthocyanin content and soluble solids rose from the first stage to the final stage of the maturity of blackberries.

The concentration of plant polyphenols can demonstrate differences according to the growing and maturity environment [102]. In a study, red raspberries were cultivated in several conditions by changing temperature and light intensity. Blue light was chosen as the best environment in which to obtain maximum polyphenol contents, especially flavan-3-ol derivatives of red raspberries. In another study, the diverse maturity stage at the harvest of gariguette strawberries affects the bioactive compounds, especially polyphenols [103]. These maturity stages were chosen as the turning stage and are fully ripe. At the end of the study, fully ripe harvested food had more polyphenols, especially hydroxycinnamic acids, than the turning stage. Additionally, several bioactive compounds of the strawberries, including vitamin C, organic acids, and volatiles, were detected in divergent amounts by different maturity stages.

Despite using the same plant material, the polyphenols vary depending on the growing region [104]. In a study, ripened wild strawberry (*Arbutus unedo* L.) was used to determine the effects of different growing regions on the polyphenolic ingredients [104]. The strawberries were taken from three forests: Achakar, Qsar Kbir, and Chaoun-Qalaa. As a result of this study, some polyphenols, including tannins, anthocyanins, catechic tannins, gallic tannins, coumarins, and anthraquinones, did not show any differentiation related to growing regions. However, quinone polyphenol exhibited that the strawberry obtained from the Achakar forest did not have this polyphenol compared to other strawberries from the Qsar Kbir and Chaoun-Qalaa forests. Also, the strawberries’ total polyphenol, flavonoid, anthocyanin, tannin, and antioxidant activity were different across growing regions. The best results were demonstrated with the strawberries obtained from the Chaoun-Qalaa forest when paying attention to the total polyphenol content and flavonoid content, with Achakar forest strawberries when paying attention to antioxidant activity and tannin content and with partial Qsar Kbir forest strawberries dealing with the anthocyanin content of the strawberries. In another study, strawberry trees obtained from five various areas containing Chefchaouen, Moulay Driss Zerhoun, Laanoucer, El Ksiba, and Tahnaout were examined based on their antioxidant activity, organic acid, and phenolic composition [80]. As a result, the total phenols were determined in the trees that were taken from the Laanoucer, Moulay Driss Zerhoun, Chefchaouen, Tahnaout, and El Ksiba, respectively. Total flavonoids were determined from the trees in the Tahnaout, Moulay Driss Zerhoun, Laanoucer, Chefchaouen, and El Ksiba, respectively. The total anthocyanins were determined from the trees in Moulay Driss Zerhoun, Tahnaout, Chefchaouen, Laanoucer, and El Ksiba, respectively. Also, antioxidant activity was significantly high in the tree obtained from the Moulay Driss Zerhoun region.

The ingredients of the foods, especially polyphenols, change according to seasons, which have different environmental conditions [105]. The biological activity, aroma, and polyphenols of white strawberries changed according to the climate conditions and growth location of the fruit [105]. Also, the growing year of foods is an effective parameter for obtaining polyphenols [91]. Researchers used strawberries harvested in 2014 and 2015 to extract the polyphenols. The total anthocyanin and phenolic content of the strawberries harvested in 2015 was higher than that of 2014. Still, the lower content of cyanidin-based forms was obtained in strawberries harvested in 2015.

The food types demonstrate differentiation in terms of their polyphenol ingredients compared with each other [91]. Several cultivars of the strawberry were used to obtain the polyphenols in a study [91]. According to the results, each cultivar gave a different total phenolic content, total anthocyanin content, and antioxidant activity, despite all cultivars being strawberries. Additionally, 11 different Spanish almonds with specific genotypes were extracted for their polyphenols in another study [106]. In conclusion, the number and types of polyphenols, including (+)-catechin, (−)-epicatechin, isorhamnetin-3-O-glucoside, kaempferol-3-*O*-rutinoside, isorhamentin-3-*O*-rutinoside, sum Flavan-3-ols, and sum Flavanols of the Spanish almonds were different from each other due to their divergent genotypes. In another study, various polyphenols were obtained from several blackberry cultivars (*Rubus* spp.), including Cheste, Triple Crown, Navaho, Loch Ness, Thornfree, and Ouachita [107]. As a result, several anthocyanins, including cyanidin-3-glucoside, cyanidin-3-O-arabinoside, cyanidin-3-O-(malonyl)glucoside, cyanidin-3-O-(dioxalyl)glucoside, and cyanidin-3-rutinoside, exhibited alternative concentrations in each cultivar when compared with others. According to these results, several blackberry (*Rubus* spp.) fruit cultivars could have different types and concentrations of polyphenols.

The polyphenolic compounds can vary with extraction types, including ultrasound-assisted and conventional solvent extractions [78]. Turmeric (*Curcuma longa*) was extracted using divergent extraction methods, including ultrasound-assisted and conventional solvent extractions [78]. In conclusion, the concentration of gallic acid, protocatechuic acid, catechin, chlorogenic acid, epicatechin, ferulic acid, coumarin, and rutin was detected more in ultrasound-assisted extraction than in conventional solvent extraction, even though some of these polyphenols were not detected in conventional solvent extraction. Despite these factors, some polyphenols, including curcumin, myricetin, cinnamic acid, genistein, and quercetin, were determined in ultrasound-assisted extraction to be no more or less than in conventional solvent extraction. In another study, garden blackberries (*Rubus fruticosus* L.) were extracted with different extraction solvents, including 80% (*v*/*v*) ethanol, 70% (*v*/*v*) acetone + 2% (*v*/*v*) acetic acid, 60% (*v*/*v*) methanol + 3% (*v*/*v*) formic acid, and 90% (*v*/*v*) acetonitrile + 10% (*v*/*v*) 6 molar HCl [108]. At the end of the study, the maximum anthocyanin, flavonoid, and polyphenol contents of garden blackberries were extracted with 90% (*v*/*v*) acetonitrile + 10% (*v*/*v*) 6 molar HCl as extractant. The ethanol and other solvents can be toxic for food processing; however, this study focused on obtaining most polyphenols from food not for use in food processing [97]. If manufacturers want to use polyphenol-extracted plant materials in food processing, they can use green solvents [97]. The green solvent can be 50% ethanol, 50% glycerol, and 100% distilled water, all acidified with 1% citric acid or 1% formic acid for anthocyanin and total phenolic content extraction from lyophilised chokeberry pomace used in food production.

Foods can have different sizes and shapes, despite being the same food, and the size of foods is relevant to the absorption of polyphenols [77]. Clove and thyme were prepared as whole foods, and powdered foods and different extracts were used to obtain polyphenols in the study [77]. As a result, whole and powdered versions of these foods showed differentiation in terms of the phenolic and flavonoid compounds. Generally, the powder of these foods had more phenolic and flavonoid compounds than the whole. Also, extracts were effective in the concentration of phenolic and flavonoid compounds in the foods. While ethanolic extract was the best extraction to obtain these compounds from thyme, essential oil extract was the best to obtain ethanolic extract, aqua extract, and essential oil extracts from clove. These differences in the yield of polyphenols are generally sourced from the differences in extraction methods, not plant materials.

The germination day is another parameter to obtain maximum polyphenolic ingredients [106]. Separately, 10 days of germinated Egyptian chia seeds (*Salvia hispanica* L.) were watched to determine the importance of the germination day on the polyphenol content [72]. The total phenolic and flavonoid contents increased from 0 to 7 germination days; however, these contents decreased from 7 to 10 days.

## 4. Application of Polyphenols in the Food Industry

**Figure 3:**
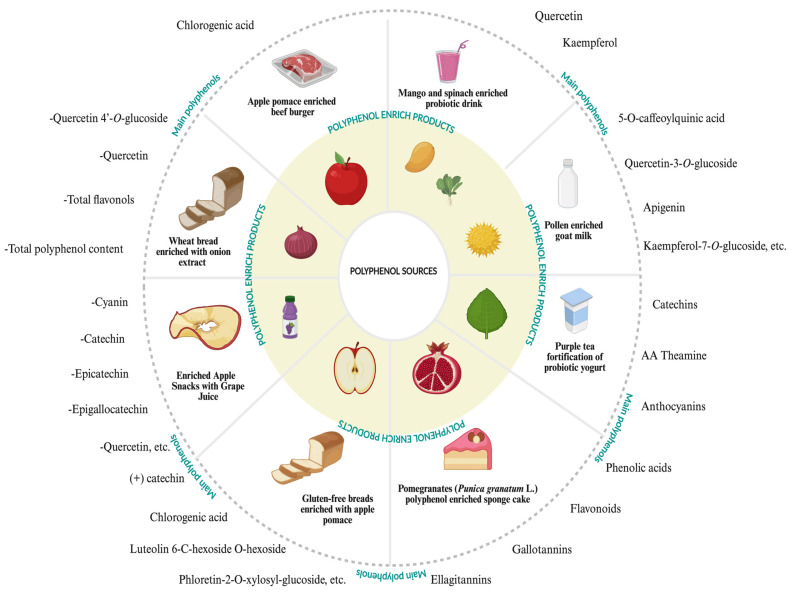
Functional foods containing common polyphenol sources and the main types of polyphenols they contain (information has been taken from Google Scholar).

Polyphenols in plant-based foods are used in the food industry to enhance functional foods, food preservation and stability, and food packaging (Figure 3) [109,110]. Also, they increase the shelf life of foods and beverages, or functional food processing, and the bioaccessibility of food ingredients [111,112]. The effects of polyphenols are degraded by digestion, and the effect of the digestion on antioxidant activity and bioaccessibility of polyphenols was examined [113]. This accessibility problem could be prevented by several methods, including microencapsulation or encapsulation [40,114]. Also, the nano-chitosan and chitosan coatings were used to preserve the polyphenol ingredients of the foods [115]. These protective studies, which are applied to the polyphenols of foods, can be used in functional food and polyphenol-rich product manufacturing due to the positive effect of these methods on the preservation of polyphenols [116]. Also, polyphenols were used for the packaging and preservation of food, including strawberries, besides their own highly bioactive ingredients [42]. Additionally, for the industrial usage of the food polyphenols, various technologies, including freezing, thermal treatments, and high-pressure processing, were used, and the effects of the technologies on the apples and strawberries were determined [117]. Different technologies provide several results that differ according to the fruits and their bioactive ingredients, especially polyphenols. As a result of the study, these technologies could be used in the food industry to produce effective foods. Recent studies also demonstrated that date fruits are a valuable polyphenol source in functional food production [118]. Their polyphenolic contents, including ferulic, *p*-coumaric, protocatechuic, gallic, syringic, *p*-hydroxybenzoic, vanillic, and salicylic acids as phenolic acids and rutin, naringenin, luteolin, catechin, and epicatechin as flavonoids, were identified by high performance liquid chromatography–electrospray ionization–mass spectrometry. Their polyphenols increased the antioxidant activity of the produced functional foods [119].

### Functional Foods and Polyphenol-Enriched Products

Polyphenols have several health-promoting properties, including anti-aging, anti-inflammatory, and anti-cancer effects [120,121,122]. For these reasons, functional foods and polyphenol-enriched products are manufactured in diets (Table 2) [46,123]. In a study, raspberry polyphenols were added to probiotic dairy products fortified with oat bran, and a functional food was produced for effective diets [123]. Also, this study examined and reported the effect of oat bran and probiotics on polyphenolic ingredients. Antioxidant activity and polyphenols were preserved at the specific storage conditions determined in the study. In another functional food production study, fermented mango (*Mangifera indica*) and spinach flour (*Amaranthus*) were used to enrich probiotic drinks in terms of polyphenols [124]. *Lactobacillus paracasei* was incubated for 60 h in anaerobic conditions at a temperature of 30–32 °C. The polyphenol ingredients of the functional drink were determined by high-performance liquid chromatography. As a result of the study, the anti-diabetic properties, including the ability to improve the lipid profile and stabilize the blood sugar fluctuation in the functional drink, were searched for and determined

Different compositions of the added material to polyphenols are important for maximum accessibility to these in functional foods or polyphenol-enriched products [126]. In a research study, several milk compositions, including full-fat, semi-skimmed, skimmed, or high-protein milk, were used to produce functional sport-supported beverages enhanced with blackberry polyphenols [126]. Sport-supported beverages are functional beverages designed to increase physical performance and to support after exercise. The addition of milk preserved the polyphenols during digestion and increased their bioaccessibility due to the preservation ability of the milk fat from degradation. Full fat exhibited the best preservation at the end of the study with in vitro digestion. In another study about milk and polyphenol interaction, bioaccessibility and antioxidant activity were increased in digestion, which was determined with an in vitro study [41]. Blackcurrant polyphenols were bonded with milk proteins, including whey protein and especially casein, to improve their bioaccessibility and antioxidant activity. This interaction showed the best bioaccessibility and antioxidant activity of polyphenols compared to alone milk and blackcurrant digestion. Also, the polyphenol–protein interaction increased the resistance of the milk.

Functional yogurts can also be produced with polyphenols to enhance the health-promoting activity and resistance of yogurt [125]. Firstly, yogurt was fermented with probiotic culture and purple tea polyphenols were extracted. The polyphenols did not have any effect on the probiotics in yogurt. However, they increased the beneficial bacteria, including *Lactobacillus* and *Bifidobacterium genera*, and decreased the pathogens, including *Staphylococcus*, *Helicobacter*, *Mycoplasma*, and *Aerococcus*, in the gut microbiota, showing that the polyphenols have prebiotic activity.

Several by-products have resulted from the food industry [130]. These should be used in alternative ways, including functional food production [132]. Grape seeds are by-products; muffins produced with different flours were enriched with grape seed extract to enhance the nutritional value of the products [132]. Whole wheat flour, whole siyez wheat flour, and whole oat flour were used to produce muffins, and better antioxidant activity and total phenolic content were best used in the muffins prepared with whole oat flour.

Blackberry juice was encapsulated with apple fibers to carry the polyphenols in a study [127]. The effect of different amounts of fiber on polyphenols was detected, and a high amount of fiber demonstrated a negative effect on the polyphenol content and preservation. The functional food was produced with apple fibers and blackberries. The antioxidant activity and inhibition of α-amylase were determined in the food. In another study, pomegranate (*Punica granatum* L.) peel extract was used as a natural polyphenol source to enrich the sponge cake [128]. In the analysis, pomegranate peel extract showed high yeast α-glucosidase and α-amylase inhibitory effects, and a decrease in the GI and starch hydrolysis index of the cake was determined. Also, the digestibility of the cake increased with polyphenol enrichment in this study.

The gluten-free bread is a functional food, especially in the diet; bread can be improved with polyphenol-rich foods [129]. Gluten-free bread was prepared with apple pomace to enhance the nutritional value of the food [129]. The identification of the polyphenols was applied to the bread, and several types of flavonols, phenolic acids, flavan-3-ols, and dihydrochalcones were determined, and the gluten-free bread was improved in terms of antioxidant activity and polyphenolic contents.

## 5. By-Product, Including High Polyphenol Valorization in the Food Industry

Food by-products contain a high number of polyphenols, depending on the initial plant materials used, and can be valorized based on an adequate industrial foundation in order to manufacture several types of functional food [129,133]. These functional foods are chosen by people because they are important for their health [134,135].

Various functional foods have been presented in the food industry, including dietary meat, bread, and cake production [23,136,137]. In a study, gluten-free breadsticks were enriched with olive by-products containing high polyphenol ingredients [23]. Olive leaf extract was prepared by an ultrasound-assisted method, and olive mill wastewater extract was prepared by another method, including acidic and hexane washing. Olive leaf extract was stored at −20 °C after freeze-drying, while olive mill wastewater extract was stored at 4 °C as a liquid. Antioxidant activity and total phenolic content of olive by-product extract flours were determined before the manufacturing of the functional food. Olive leaf extract had more total phenolics and antioxidant activity compared to olive mill wastewater extract. Additionally, similar results were obtained after baking when compared to the breadsticks they prepared separately with olive leaf extract and olive mill wastewater extract flours. The flours were used in baking breadsticks, and their water activity, moisture level, color, and textural properties were determined after production. At the end of the study, a functional food with high polyphenol ingredients, shelf life, preserved color, and textural properties was obtained. Instead of the best results coming from olive leaf extract, it had a slightly lower shelf life compared to other olive by-products. All of these results showed that olive leaf extract was more usable than olive mill wastewater extract in functional breadstick production. In a similar study, another olive by-product was used to prepare the enriched wheat bread production [138]. Fermented green olive pulp was used for the fortification of the wheat bread, and after the manufacturing of the bread, bioactive compounds, antioxidant activities, phenolic compounds, fatty acids, and sensory properties were measured. Experiments showed that fortified wheat bread had high phenolic ingredients, especially gallic acid and rutin, and antioxidant activity and protection of textural properties when compared with the control sample, which had not applied any fortification. In addition, functional biscuits were produced, valorizing the date palm by-products [139]. The addition of their by-products acquired storage stability and rich polyphenolic power for the biscuits.

Furthermore, functional food bioactive ingredients and activities can change with the percentage of added by-products [140]. Tomato by-products, including seeds and skins, were used to enrich bread in terms of carotenoids, ascorbic acid, total phenolic content, antioxidant activity, and mineral and trace element contents [140]. First, these by-product extracts were dehydrated, and 6% and 10% tomato wastes were used for fortifying the bread. Different percentages affected the results of the bread in terms of the bioactive properties. The lower percentage of the by-products gave more effective results, especially in lycopene and carotenoids, and also less in total phenolic ingredients. Additionally, biscuit doughs were fortified with pomegranate peel powder as a food by-product material [141]. Similar to the previous study, different concentrations of food by-products were used, and different results were obtained. The high-performance liquid chromatography method was used to determine the polyphenolic ingredients, and remarkable tannin types were determined in the study. At the end of the study, experiments showed that fortification with pomegranate peel powder contributed to biscuit doughs in terms of the bioactive ingredients, especially phenolic contents, antioxidant activity, and textural and sensorial properties.

*Hodgsonia heteroclita* oilseed cake, which was enriched with oilseed by-products, was examined in the shape of powder and in terms of the antioxidant activity and presentation of bioactive compounds, including polyphenols [136]. In this study, the health benefits and antioxidant activities of the fortified cake were determined. 4-Hydroxybenzoic acid and ferulic acid were particularly identified by liquid chromatography–electrospray ionization tandem mass spectrometry analysis. The crucial enzyme inhibitory activities, including lipase in obesity, α-amylase, α-glucosidase, and dipeptidyl peptidase IV in diabetes activities, are important for the human body and have been studied. In conclusion, oil seeds contributed to the bioactive ingredients, enzyme inhibition, and antioxidant activities of the cake.

## 6. Conclusions

Plant-based foods have been used for nutrition for several, even more than millions, of years. They are rich in bioactive components that have several effects and activities, including anti-cancer effects, anti-tumor effects, anti-inflammatory effects, cardiometabolic risk management, antimicrobial effects, immunomodulatory activity, antioxidant activity, and antiradical activity. The most common bioactive compounds of plants are polyphenols, and they have been used for several reasons to improve human health and food quality. They also have several subgroups, including anthocyanins and flavonols, and several types of polyphenols, including rutin and quercetin, are found in food. Moreover, the amount and type of polyphenols demonstrate various results according to the type of food, and some factors of polyphenols differentiate the polyphenols, including germination day, harvested year, ripening, and growing region. Additionally, the effects of polyphenols on diseases and gut microbiota are investigated in vivo, in vitro, or in other experiments. Furthermore, the stability and bioaccessibility of the polyphenols were searched for and detected, and alternative ways, including encapsulation and coating, were enhanced. The extraction of polyphenols is a basic process; however, it exhibits inconsistencies for several characteristic processes, including extractant type. In addition, the characterization of polyphenols is applied with chromatographic methods, which are discrepancies according to the types of food and the region in which polyphenols will be obtained, including the seeds or skin of food products. Additionally, polyphenols are utilized in the food industry, particularly in the production of functional foods, where polyphenol-rich foods and their by-products serve as a source of functional food enrichment. Moreover, polyphenol-enhanced functional foods offer high nutritional value for consumption in various dietary fields. The polyphenols and their usage potential in the food industry, especially functional food production, are suggested by various research papers cited in this review article. The studies about polyphenols and their use in the food industry are still being carried out. Furthermore, this review article summarizes the studies about polyphenols and their use in the food industry in recent years, as different from other review articles.

## Figures and Tables

**Table 1:** Polyphenol extraction methods and properties of polyphenol food sources.

Polyphenol Sources	Polyphenols	Extraction Methods	Detection Methods	Studied Effect or Property of Food Polyphenols	References
**Walnut**	-Ellagic acid -Strictinin -3-Methoxy-5,7,3′,4′-tetrahydroxy-flavon -Gallic acid -Ellagic acid pentoside, etc.	-100% hexane (1:10 *w*/*v*) as extractant in solid-phase extraction.	-Folin–Ciocalteu method for total phenolic content -Reverse-phase high-performance liquid chromatography and high-resolution Fourier transform mass spectrometry to identify polyphenols	Exhibit inhibition of human intestinal glucose transport, human α-glucosidase activities, and human salivary and pancreatic α-amylases.	[57]
**Blackberry**	-Anthocyanins -Cyanidin-3-*O*-glucoside -Cyanidin-3-*O*-rutinoside -Ellagitannins -Catechins etc.	-70% acetone as extractant in an in-house solid–liquid extraction method.	-Flash chromatography and mass spectrometry	Exhibit antioxidant activity.	[58]
**Black raspberry**	-Cyanidin-3 -5-O-diglucoside -Pedunculagin/casuariin -Caffeoyl-hexoside -Sanguiin H-6, etc.	-The divergent percentage of Methanol, acetone, and ethanol with 30% water as extractant	-Ultra-high-performance liquid chromatography quadrupole time-of-flight mass spectrometry/mass spectrometry	Exhibit a health-promoting effect on gut microbiota.	[59]
**Red Onion Peels**	-Benzoic acid -Rosmarinic acid -Quercetin -Rutin -Pyrogallol -Quercetin -Quercetin derivatives -ρ-Coumaric acid	-Ultrasound- and enzymatic-assisted extractions with 80% ethanol and citrate buffer with enzyme.	-High-performance liquid chromatography analysis	Exhibit antioxidant activity.	[60]
**Lentil (*Lens culinaris*)**	-Total phenolic compounds	-Acetone: water (80:20 *v*/*v*) as an extractant in acetone extraction.	-Folin–Ciocalteu method for total phenolic content	Exhibit antioxidant activity.	[61]
**Raspberry Flower Petals**	-(+)-catechin -(−)-epicatechin -Procyanidin B4 -Procyanidin C3 -Sanguiin H-6 -Lambertianin C -(−)-epicatechin-3,5-di-O-gallate -Kaempferol-7-O-glucoside -Naringenin-7-O-glucoside, etc.	-Methanol as an extractant in LC-grade methanol extraction.	-High-performance liquid chromatography and liquid chromatography–mass spectrometry to identify polyphenols -The Folin–Ciocalteu method for total polyphenol content	Exhibit antioxidant activity, lipid peroxidation inhibitory activity, and inhibitory activity against cervical cancer (HeLa S3) cells.	[12]
**Chinese raspberry**	-Gallic acid -Epicatechin -Ellagic acid -Rutin -Quercetin 3-O-glucoside -Avicularin -Kaempferol-7-O-glucuronide -Quercetin-7-O-glucuronide, etc.	-70% (*v*/*v*) ethanol solution as extractant in ultrasonic extraction.	-High-performance liquid chromatography to identify polyphenols -A method that used acidic methanol (1% [*v*/*v*] HCl) for total anthocyanin content -The Al(NO_3_)_3_ - NaOH assay for total flavonoid content -Folin–Ciocalteu’s method for total polyphenol content	Exhibit antioxidant activity and cytotoxic effect.	[62]
**Roasted hazelnut skin**	-Gallic acid -Protocatechuic acid -Catechin -Epicatechin -Quercetin	-Pure ethanol as an extractant in cold extraction under magnetic stirring.	-Spectrophotometry for total polyphenolic content -High-performance liquid chromatography to identify polyphenols	Exhibit antioxidant activity.	[63]
**Welsh Onion (*Allium fistulosum*) leaves**	-Total phenolic content -Total anthocyanin content -Especially Cyanidin and quercetin-3-glucoside	-70% *v*/*v* ethanol as extractant in ultrasound-assisted extraction.	-Ultra-high-performance liquid chromatography–electrospray positive ionization positive mode - Orbitrap mass spectrometry analysis	Exhibit antioxidant activity.	[64]
**Pecan**	-Total phenolic content	-Acetone/deionized water/acetic acid (70:29.5:0.5, *v*/*v*/*v*) at a ratio of 6:10 (*w*/*v*) as extractant in an extraction method using an ASE 200 accelerated solvent extractor.	-Folin–Ciocalteu’s method for total phenolic content	Exhibit α-amylase inhibitory, and α-glucosidase inhibitory effects during starch digestion.	[65]
**Star anise (*Illicium verum*)**	-Gallic acid -4-Hydroxybenzoic acid -Catechin -Chlorogenic acid -Caffeic acid -Syringic acid -Vanillic acid -*p*-Coumaric acid -Salicylic acid -Rutin, etc.	-Distilled water as an extractant	-High-performance liquid chromatography to identify polyphenols	Exhibit antioxidant, anti-obesity, and hypolipidemic effects.	[66]
**Domestic Norwegian Apple (*Malus* × *domestica* Borkh.)**	-Chlorogenic acid -3-*O*-caffeoylquinic acid -Phlorizin -Quercetin 3-*O*-glucoside -Quercetin 3-*O*-rhamnoside -5-*O*-caffeoylquinic acid-phloretin	-Acidified methanol/water solution (70/30 with 0.1% hydrochloric acid to pH 2) as extractant in ultrasound-assisted extraction.	-Ultra-high-performance liquid chromatography system-linear trap quadrupole to identify polyphenols	Exhibit antioxidant activity.	[49]
**Blueberry (*Vaccinium* spp.)**	-Delphinidin-3-glucoside -Quercetin 3-O-galactoside -Pelargonidin-3-O-galactoside -Malvidin-3-O-glucose -Phenylpropanoid com-pound chlorogenic acid isomers -Flavonoid substance epicatechin gallate -Kaempferol-3-rhamnoside	-Acidified methanol (0.3% HCl [*v*/*v*]) as the main extractant in liquid–liquid stratification extraction.	-High-performance liquid chromatography analysis and mass spectrometry	Exhibit antioxidant activity, antitumor activity, and immune function of anthocyanins.	[67]
**Black Bean (*Phaseolus vulgaris* L.)**	-Malvidin-3-glucoside -Cyanidin-3-glucoside -Delphinidin-3-glucoside -petunidin-3-O-β glucoside -Catechin -Delphinidin 3-Glucoside -Myricetin -Sinapic acid, etc.	-Ethanol-water (50:50 *v*/*v*) as an extractant in supercritical fluid extraction	-Folin–Ciocalteu’s method for polyphenols -pH differential method (AOAC Official Method 2005.02) for total anthocyanins -Electrospray ionization mass spectrometry analysis to identify phenolic compounds	Exhibit antioxidant activity and anti-aging potential.	[68]
**Chickpea hull**	-Gallic acid -Rutin, etc.	-Acetone, water, and acetic acid (70:29.5:0.5, *v*/*v*/*v*) as extractant	-Ultra-high-performance liquid chromatography to identify polyphenols	Exhibit anti-inflammatory and antioxidant properties.	[69]
**Spanish Almonds**	-(+)-Catechin -(−)-Epicatechin -Isorhamnetin-3-*O*-glucoside -Kaempferol-3-*O*-glucoside -Isorhamnetin-3-*O*-rutinoside -Sum Flavan-3-ols -Sum Flavanols	-Hydrochloric acid, water, and methanol (3.7:46.3:50, *v*/*v*/*v*) solution as the extractant	-Spectrophotometric techniques with the modified Folin–Ciocalteu method for total polyphenol determination -Zhishen, Meng Cheng, and Jianming’s method that was modified by Jahanbani-Esfahlan and Jamei for total flavonoid determination -Ribéreau-Gayon and Stonestreet for total proanthocyanidin determination	Exhibit the antioxidant activity.	[70]
**Flax (*Linum usitatissimum* L.) Seed**	-Oleocanthal -Oleuropein -Hesperetin -Ursolic acid -Amentoflavone -Quercetin-3-*O*-glucoside -Quercetin-3-*O*-glucuronic acid -Kaempferol-3-*O*-glucose -Quercetin-3-O-hexose-deoxyhexose, etc.	-70% methanol as extractant in ultrasound-assisted extraction.	-Liquid chromatography with tandem mass spectrometry analysis to identify polyphenols	Exhibit antidiabetic effect, anti-inflammatory effect, α-Amylase inhibitory activity, and α-Glucosidase inhibitory activity.	[71]
**Egyptian chia (*Salvia hispanica* L.) seeds**	-Gallic acid -Protocatechuic acid -*p*-hydroxybenzoic acid -Chlorogenic acid -Catechin -Quercetin -Apigenin -Kaempferol	-80% methanol as extractant in the extraction.	-Distilled water, NaNO2, 10% AlCl3, and 1.0 M NaOH in a method used for total flavonoid content -Folin–Ciocalteu method for total phenolic content -High-performance liquid chromatography to identify polyphenols	Exhibit antimicrobial effect and antioxidant activity.	[72]
**Oregano (*Lippia palmeri* Watts)**	-Total polyphenol content -Total flavonoid content	-Ethanol (100%) as an extractant in the extraction.	-The Folin–Ciocalteu method for total polyphenol content -The method based on aluminum chloride for total flavonoid content	Exhibit antioxidant activity and intestinal and immunobiological effects.	[73]
**Sweet basil leaves (Ocimum basilicum L.)**	-Tannins -Flavonoids	-Ethanol 70% as an extractant in the extraction.	-Phytochemical analysis to detect secondary metabolites -The Folin–Ciocalteu method for total polyphenol content -The method based on aluminum chloride for total flavonoid content	Exhibit antioxidant activity.	[74]
**Raspberry leaf**	-Quercetin -Kaempferol -Procyanidin B1 -Catechin -Epicatechin -Gallic acid -Chlorogenic acid -*p*-Coumaric acid -Protocatechuic acid -Caffeic acid, etc.	-60% ethanol as an extractant in ultrasound-assisted extraction.	-High-performance liquid chromatography–mass spectrometer to identify polyphenols	Exhibit anti-pathogen activity and intestinal health.	[2]
**Artichoke**	-Luteolin -Luteolin-O-glycoside -Luteolin-7-O-rutinoside -Apigenin, etc.	-Ethyl acetate and methanol as extractants in an extraction method.	-High-performance liquid chromatography/electrospray ionization tandem mass spectrometry	Exhibit hepatoprotective activity.	[75]
**Egg Plant** **Varieties** **and** **Spinach Varieties**	-Total phenolic content -Total flavonoid content	-Methanolic extraction by using methanol/water (80%, *v*/*v*) as an extractant	-Folin–Ciocalteu method for total phenolic content -A spectrophotometric method for the total flavonoid content	Exhibit antioxidant activity.	[76]
**Clove (*Syzygium aromaticum*) and Thyme (*Thymus vulgaris*)**	-Total phenolic content -Total flavonoid compounds	-95% ethyl alcohol as an extractant in the extraction methods	-Folin–Ciocalteu method for total phenolic compounds -Aluminum chloride colorimetric method for total flavonoid compounds	Exhibit antioxidant and antibacterial activities.	[77]
**Turmeric (*Curcuma longa*)**	-Gallic acid -Epicatechin -Protocatechuic acid -Catechin -Chlorogenic acid -Ferulic acid -Coumarin -Rutin, etc.	-Ethanol (80%) as an extractant in ultrasound-assisted and conventional solvent extraction	-The Folin–Ciocalteu method for total phenolic content -High-performance liquid chromatography to identify polyphenols	Exhibit antioxidant and antiproliferative activities.	[78]
**Strawberry**	-Pelargonidin 3-O-glucoside and Pelargonidin-derivative -Cyanidin 3-O-glucoside and cyanidin-derivative -Gallic acid	-70% ethanol as an extractant in the extraction method	-pH differential method for total monomeric anthocyanin content -High-performance liquid chromatography-diode array detection for anthocyanins -Folin–Ciocalteu method for total phenolic content	Exhibit antioxidant activity.	[79]
**Young apple**	-Procyanidin B1 -(-)-Epigallocatechin -(+)-Catechin -Procyanidin B2 -Chlorogenic acid -4-*p*-coumaroylquinic acid -(-)-Epicatechin -Caffeic acid -Quercetin, etc.	-70% ethyl alcohol solution as an extractant	-High-performance liquid chromatography to identify polyphenols -The Folin–Ciocalteu method for total polyphenol content	Exhibit α-glucosidase inhibitory effect.	[9]
**Strawberry Tree Fruits (*Arbutus unedo* L.)**	-Rutin -Cyanidin-3-glucoside -Quercetin-3-Xylosidase -Cyanidin-30.5-diglucoside -Quercetin-3-galactoside, etc.	-Acetone/water (70:30, *v*/*v*) mixture as an extractant in an extraction method uses an IKA T−18 digital Ultra-Turrax homogenizer	-High-performance liquid chromatography to identify polyphenols -The method based on aluminum chloride for total flavonoid content -Folin–Ciocalteu method for total phenol content -pH differential method for total anthocyanins	Exhibit antioxidant activities.	[80]
**Highbush blueberries**	-Total polyphenol fraction -Anthocyanin-enriched fraction -Proanthocyanidin-enriched fraction	-70% (*v*/*v*) acetone as main extractant -After acetone extraction, methanol for the total polyphenol fraction, 50% (*v*/*v*) ethanol for anthocyanin-enriched fraction, and 80% (*v*/*v*) acetone for proanthocyanidin-enriched fraction	-High-performance liquid chromatography	Exhibit antimicrobial and anti-inflammatory effects.	[81]
**Red Raspberry**	-Quercetin -Myricetin -Ellagic acid -(+)-Catechin -(−)-Epicatechin -Cyanidin 3-*O*-β-d-glucoside -Cyanidin 3-*O*-β-d-glucoside equivalent	-Acidified methanol (0.5% acetic acid) as an extractant	-High-performance liquid chromatography to identify polyphenols -Folin–Ciocalteu method for total phenolic content	Exhibit the inhibition of NLRP3 inflammasome activation.	[4]

**Table 2:** Polyphenol usage outcome discrepancies in the food industry.

Product Types	Polyphenols	Outcome	References
**Purple tea fortification of probiotic yogurt**	-Polyphenols -Catechins -AA Theamine -Anthocyanins	-The tea polyphenols did not affect the probiotics in storage -Increased the beneficial bacteria -Decreased the pathogens in gut microbiota	[125]
**Microencapsulated Asiatic Pennywort (*Centella asiatica)* fortified chocolate oat milk beverage**	-Asiatic acid -Asiaticoside -Benzoic acid -Caffeic acid -Catechin -Chlorogenic acid -Gallic acid -Kaempferol -Luteolin -Madecassic -*p*-Coumaric acid -Quercetin -Rutin	-Preserved the polyphenolic ingredients of the food	[37]
**Polyphenol enriched milk**	-Rutin -Cyanidin-3-rutinoside -Procyanidin B1 -Delphinidin-3-rutinoside -Gallic acid, etc.	-Increased the bioaccessibility and antioxidant activity of food ingredients	[41]
**Sports nutrition milk enriched with blackberry**	-Phenolics -Flavonoids -Anthocyanins	-Enhanced the bioaccessibility of the polyphenols of the blackberry and the protection of anthocyanins in digestion	[126]
**Blackberry juice with apple fibers**	-Anthocyanins -Flavanols -Phenolic acids -Dihydrochalcones	-Showed high antioxidant activity and inhibition of α-amylase enzymes	[127]
**Apple pomace enriched beef burger**	-Chlorogenic acid -Quercetin-3-*O*-glucoside -Phloridzin	-Demonstrated the high total phenol content, antioxidant activity, and antioxidant compounds, including quercetin derivatives, chlorogenic acid, and phloridzin in the enriched beef burger	[28]
**Oat bran fortified raspberry probiotic dairy drinks**	-Phenolic acids -Flavonoids -Phytic acid, etc.	-Did not cause any negative effect on the polyphenolic ingredients of functional food in storage	[123]
**Fermented mango (*Mangifera indica*) and spinach flour (*Amaranthus*) enriched probiotic drink**	-Quercetin -Kaempferol	-Improved lipid profiles -Stabilized blood sugar fluctuations so that they can be anti-diabetics	[124]
**Polyphenols enriched ice cream, yogurt, and buttermilk with black carrot (*Daucus carota* L.) concentrate**	-Total phenols -Total flavonoids -Anthocyanins	-Enhanced the mineral content (Mg and Fe), polyphenols, and antioxidant activity of dairy products	[128]
**Gluten-free breads enriched with apple pomace**	-Luteolin 6-C-hexoside *O*-hexoside -Chlorogenic acid -(+) catechin -Phloretin-2-*O*-xylosyl-glucoside, etc.	-Improved the nutritional value of the bread in terms of especially polyphenols -Demonstrated high antioxidant activity and polyphenolic ingredients	[129]
**Spent Coffee Grounds-Enriched Cookies**	-Melanoidins -Chlorogenic acid -5-caffeoylquinic acid -Phenolic acids, etc.	-Improved the polyphenolic ingredients of the food -Enhanced the bioaccessibility and antioxidant activity of the cookies	[7]
**Pollen-enriched goat milk**	-5-O-caffeoylquinic acid -Quercetin-3-*O*-glucoside -Apigenin -Kaempferol-7-*O*-glucoside, etc.	-Enhanced the antioxidant activity and bioaccessibility in digestion	[26]
**Olive leaves and olive mill wastewater-enriched gluten-free breadsticks**	-Total polyphenol content	-Demonstrated antioxidant activity and high polyphenol bioaccessibility in the breadsticks	[23]
**Grape pomace and olive pomace enriched tagliatelle pasta**	-Quercetin -Kaempferol -Delphinidin-3-*O*-glucoside -Petunidin-3-*O*-glucoside, etc.	-Improved the nutritional value of the food	[130]
**Functional beef burgers formulated with chia seeds and goji puree**	-Carotenoids -Chlorogenic acid -Caffeic acids -Quercetin -Kaempferol	-Enhanced bioaccessibility of polyphenols	[131]
**Pomegranate (*Punica granatum* L.) polyphenol-enriched sponge cake**	-Phenolic acids -Flavonoids -Gallotannins -Ellagitannins	-Enhanced the nutritional value and total phenolic ingredient -Inhibition of α-Glucosidase and α-amylase -Showed high digestibility ability	[128]
**Rye snacks enriched with seaweed extract**	-Total phenolic content	-Enriched antioxidant activity, oxidative stability ability, and preventive effect from diseases -Promoted the enhancement of the nutritional value and preservation of convenience food	[110]
**Enriched Apple Snacks with Grape Juice**	-Cyanin -Catechin -Epicatechin -Epigallocatechin -Quercetin, etc.	-Improved the polyphenolic ingredients of the product -Demonstrated high antioxidant capacity and bioaccessibility of the polyphenols in the digestion of the snacks	[5]
**Olive leaf extract-enriched taralli**	-Total Phenols -Total Flavonoids -Oleuropein, etc.	-Increased the bioaccessibility of the nutritional contents and antioxidant activity of the food	[29]
**Partially deoiled chia flour-enriched wheat pasta**	-Quinic acid -Caffeic acid -Ferulic acid -Methylquercetin, etc.	-Improved nutritional value -Enhanced bioaccessibility in digestion	[52]
**Wheat bread enriched with onion extract**	-Quercetin 4’-*O*-glucoside -Quercetin -Total flavonols -Total polyphenol content	-Demonstrated the high antioxidant activity and polyphenolic ingredients in storage	[30]
**Berry fruits-enriched pasta**	-Total polyphenol content -Anthocyanins	-Enhanced the nutritional value, bioaccessibility, antioxidant activity, and bioavailability of the pasta	[53]

## Data Availability

Not applicable.
